# Characterization of alternative splicing events and prognostic signatures in breast cancer

**DOI:** 10.1186/s12885-021-08305-6

**Published:** 2021-05-22

**Authors:** Pihua Han, Jingjun Zhu, Guang Feng, Zizhang Wang, Yanni Ding

**Affiliations:** 1 Breast Disease Center, Shaanxi Provincial Cancer Hospital, Xi’an City, 710000 Shaan Xi Province China; 2 Department of Breast Surgery, Baotou Tumor Hospital, Inner Mongolia Autonomous Region, Baotou, 014030 China; 3 The Third Department of Burns and Plastic Surgery and Center of Wound Repair, the Fourth Medical Center of PLA General Hospital, Beijing, 100048 China; 4 Department of Head and Neck Surgery, Shaanxi Provincial Cancer Hospital, Xi’an City, 710000 Shaan Xi Province China

**Keywords:** Alternative splicing, Breast cancer, The Cancer genome atlas, Prognosis

## Abstract

**Background:**

Breast cancer (BRCA) is one of the most common cancers worldwide. Abnormal alternative splicing (AS) frequently observed in cancers. This study aims to demonstrate AS events and signatures that might serve as prognostic indicators for BRCA.

**Methods:**

Original data for all seven types of splice events were obtained from TCGA SpliceSeq database. RNA-seq and clinical data of BRCA cohorts were downloaded from TCGA database. Survival-associated AS events in BRCA were analyzed by univariate COX proportional hazards regression model. Prognostic signatures were constructed for prognosis prediction in patients with BRCA based on survival-associated AS events. Pearson correlation analysis was performed to measure the correlation between the expression of splicing factors (SFs) and the percent spliced in (PSI) values of AS events. Gene ontology (GO) and Kyoto Encyclopedia of Genes and Genomes (KEGG) were conducted to demonstrate pathways in which survival-associated AS event is enriched.

**Results:**

A total of 45,421 AS events in 21,232 genes were identified. Among them, 1121 AS events in 931 genes significantly correlated with survival for BRCA. The established AS prognostic signatures of seven types could accurately predict BRCA prognosis. The comprehensive AS signature could serve as independent prognostic factor for BRCA. A SF-AS regulatory network was therefore established based on the correlation between the expression levels of SFs and PSI values of AS events.

**Conclusions:**

This study revealed survival-associated AS events and signatures that may help predict the survival outcomes of patients with BRCA. Additionally, the constructed SF-AS networks in BRCA can reveal the underlying regulatory mechanisms in BRCA.

**Supplementary Information:**

The online version contains supplementary material available at 10.1186/s12885-021-08305-6.

## Background

BRCA ranks among the top most common female malignancies in China and worldwide [1, 2]. BRCA is treated with a multidisciplinary approach including surgical resection, chemotherapy, hormonotherapy, molecular targeting treatments, and radiotherapy [3]. In the past three decades, breast cancer death rates decreased by 39%, which translates to more than 300,000 averted breast cancer deaths in the United States [4]. However, the heterogeneity and complexity of BRCA still led to poor prognosis in patients with certain types of BRCA. Thus, it is urgent to identify the potential molecular mechanisms underlying BRCA and to improve the prognosis of BRCA patients.

Alternative splicing (AS) is an important post-transcriptional process through which multiple transcripts are generated from a single gene [5]. Dysregulation of AS is known to be implicated in multiple human malignancies [6, 7]. There are seven types of AS events including exon skip (ES), alternate donor site (AD), alternate acceptor site (AA), mutually exclusive exon (ME), alternate terminator (AT), alternate promoter (AP), and retained intron (RI). Increasing evidence indicates that AS is related to cancer development and metastasis [8–11]. In addition, AS events were reported to serve as prognostic predictors for multiple types of cancer [12].

Advances in the next-generation sequencing technologies have pushed forward cancer epigenetic researches. SpliceSeq [13] database provides AS profiles across 33 tumors and enables researchers to study the global profiling of AS events in most predominant human malignant tumors.

Our study aimed to identify AS events in BRCA and its prognostic significance in BRCA patients using data downloaded from TCGA database. AS events that significantly associated with prognosis of BRCA were identified, and AS prognostic signatures based on AS events were then generated. Moreover, a SF-AS regulatory network was also established to reveal the underlying correlations among SFs and AS in BRCA.

## Methods

### AS data download and process

The percent spliced in (PSI) value was calculated and processed by SpliceSeq to quantify AS events. The PSI value indicates the inclusion of a transcript element divided by the total number of reads for that AS event. PSI values range from 0 to 100%, which indicates a shift percentage in AS events. AS events with PSI value of larger than 75% in were downloaded from SpliceSeq database. An UpSet plot was created to show the intersection and distribution among all types of AS. Then clinical data of BRCA patients were obtained from TCGA-BRCA database. The primary endpoint in our study is overall survival (OS).

### Survival analysis

Patients with an OS of less than 90 days were excluded. The follow-up periods of the BRCA cohort ranged from 91 to 8605 days. Univariate proportional hazards regression model was performed to evaluate the correlation between the PSI value of each AS event and prognosis of BRCA patients.

### Construction of prognostic signatures

A least absolute shrinkage and selection operator (LASSO) analysis to develop prognostic signatures based on the top 20 most significant AS events selected from the univariate Cox analysis. The coefficients and partial likelihood deviance were also calculated in LASSO analysis. The prognostic AS signatures were generated by multiplying the PSI values of AS events with the coefficients assigned by LASSO analysis. Then AS prognostic signatures along with multiple clinical parameters were included into multivariable Cox regression analysis to identify independent predictors. A time-dependent receiver-operator characteristic (ROC) curve was used to evaluate the prognostic prediction efficacy of the AS signatures. The risk score of each AS event was calculated to assess the performance of the prognostic signatures. Kaplan-Meier survival analysis with log-rank test was performed to evaluate the prognostic difference between high- and low-risk groups.

### SF-AS regulatory network

A list of four hundred and four SFs was obtained from the study of Seiler et al. (Table S3) [14]. The expression data of SFs were downloaded from TCGA-BRCA dataset. Correlations among the expression of SFs and the PSI values of prognostic AS events were assessed by Pearson’s correlation analysis. SFs-AS interactomes with a correlation coefficient more than 0.5 and *P* value less than 0.05 were selected to create the SF-AS network by Cytoscape software.

### Functional annotation

KEGG and GO analysis were conducted to investigate the functional categories of genes with prognostic AS events by the package “clusterProfiler” in R software.

## Results

### Landscape of AS events in BRCA

**Fig. 1:**
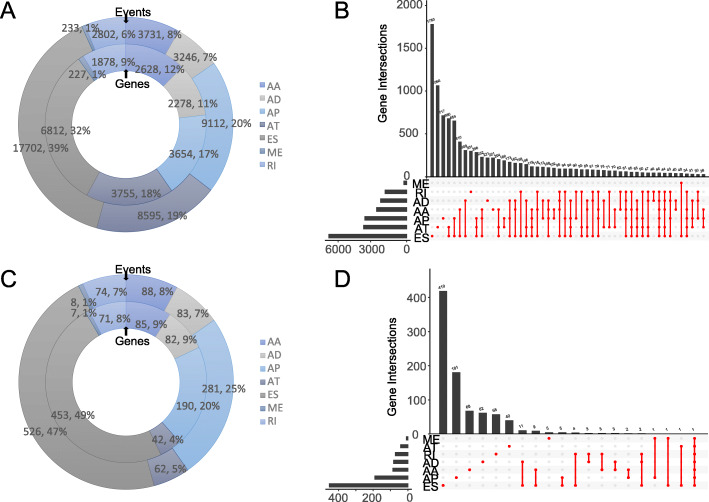
Overview of alternative splicing (AS) events and prognostic AS events in BRCA. **a** Numbers and percentages of events and corresponding genes in seven types of AS; **b** UpSet plots showing the intersection of seven types of AS events; **c** Numbers and percentages of prognosis- associated AS events and corresponding genes; **d** UpSet plots showing the intersection of prognostic AS events

TCGA splice-seq data and clinical information in TCGA-BRCA project were downloaded, and 1098 patients were enrolled in this study. In total, 45,421 AS events in 21,232 genes were identified in BRCA, suggesting that AS is a common biological process in BRCA. To be specific, 3731 AA events in 2628 genes, 3246 AD events in 2278 genes, 9112 AP events in 3654 genes, 8595 AT events in 3755 genes, 17,702 ES events in 6812 genes, 233 ME events in 227 genes, and 2802 RI events in 1878 genes were observed in preliminary analysis (Fig. 1a). Figure 1b showed the distribution and intersection among seven types of AS. ES was the predominant type, and one gene may have multiple types of AS.

### Prognostic AS events

The differential AS events between normal and BRCA tissues were identified using “limma” package in R software and demonstrated with a volcano plot. However, no significant difference of AS event was detected (Table S1). In addition, the differential expressed genes between normal and BRCA tissues were analyzed. In comparison with normal tissue, there are 57 genes upregulated and 215 genes downregulated in BRCA tissues (Table S6). A univariate Cox analysis was performed to evaluate the prognostic significance of AS events in BRCA patients. Results suggested that 1121 AS events in 931 genes were significantly correlated with the prognosis of patients with BRCA (Table S2). Specifically, 88 AA events in 85 genes, 83 AD events in 82 genes, 281 AP events in 190 genes, 62 AT events in 42 genes, 526 ES events in 453 genes, 8 ME events in 7 genes, and 74 RI events in 71 genes were identified as prognostic AS events (Fig. 1c). Additionally, one gene could present multiple AS events that were significantly associated with the OS of patients with BRCA. Figure 1d showed that ES was also the predominant AS type. To verify if a particular splicing-pattern is more enriched of prognostic isoforms, we calculate the enrichment ratio for each type of AS by divide the number of prognostic AS events by the number of overall AS events of each AS type. The ratios of AA, AD, AP, AT, ES, ME, and RI are 2.36, 2.56, 3.08, 0.72, 2.97, 3.43, and 2.64% (Table S7). With the exception of AT, which is lowest (0.72%), there was no significant difference among the ratios of the rest of 6 types of AS (2.36-3.43%). ES does not have a significantly higher ratio than other types of AS.

### Prognostic AS signatures

**Fig. 2:**
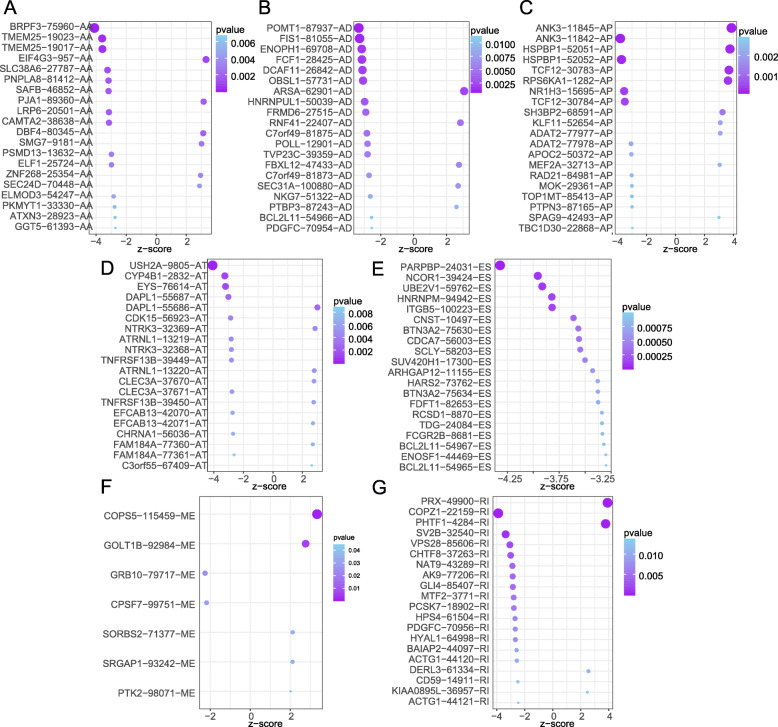
The top 20 most significant AS events in BRCA. **a** alternate acceptor, **b** alternate donor sites, **c** alternate promoters, **d** alternate terminators, **e** exon skips, **f** mutually exclusive exons, and **g** retained introns

**Fig. 3:**
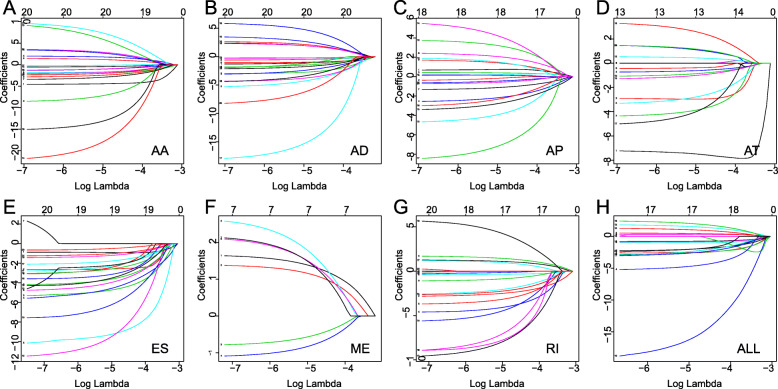
Construction of prognostic signatures based on LASSO COX analysis. Each curve in the figure represents the trajectory of each independent variable coefficient. The vertical axis is the value of the coefficient, the lower horizontal axis is log2-Lambda value, and the upper horizontal axis is the number of non-zero coefficients in the model on each scale. The small serial numbers before each curve in the box were used to mark each variable. Each colored line represents the value taken by a different coefficient in the model. Lambda is the weight given to the regularization term, so as lambda approaches zero, the loss function of the model approaches the OLS loss function. When lambda is very small (leftmost), the LASSO solution should be very close to the OLS solution, and all coefficients are in the model. As lambda grows (from left to right), the regularization term has greater effect, more and more coefficients will be zero valued and fewer variables in the model remain. **a** alternate acceptor, **b** alternate donor sites, **c** alternate promoters, **d** alternate terminators, **e** exon skips, **f** mutually exclusive exons, **g** retained introns, and **h** comprehensive signature

**Fig. 4:**
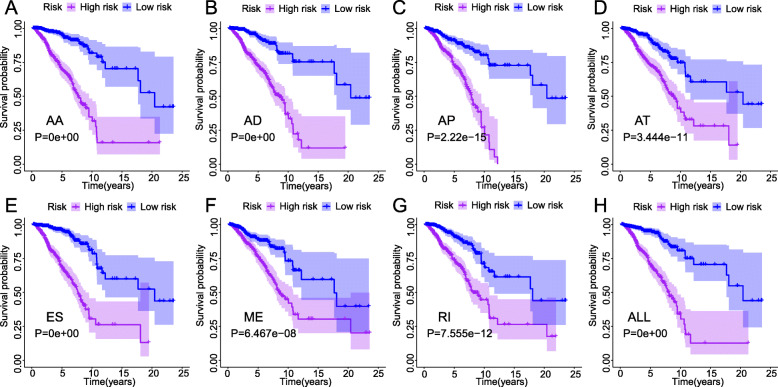
Kaplan-Meier curves of high risk (purple) and low risk (blue) BRCA patients according to eight prognostic signatures. **a** alternate acceptor, **b** alternate donor sites, **c** alternate promoters, **d** alternate terminators, **e** exon skips, **f** mutually exclusive exons, **g** retained introns, and **h** comprehensive signature

**Fig. 5:**
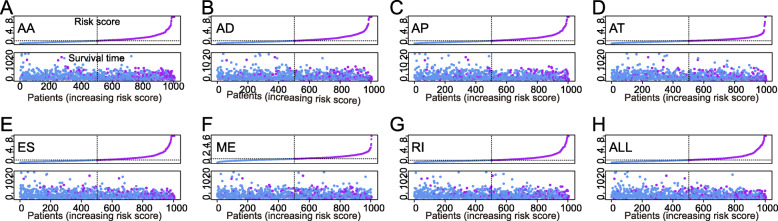
The risk scores and distribution of survival time of eight signatures in patients with BRCA. **a** alternate acceptor, **b** alternate donor sites, **c** alternate promoters, **d** alternate terminators, **e** exon skips, **f** mutually exclusive exons, **g** retained introns, and **h** comprehensive signature. Upper plot: risk score; Lower plot: survival time distribution. Purple dots: high risk; blue dots: low risk

**Fig. 6:**
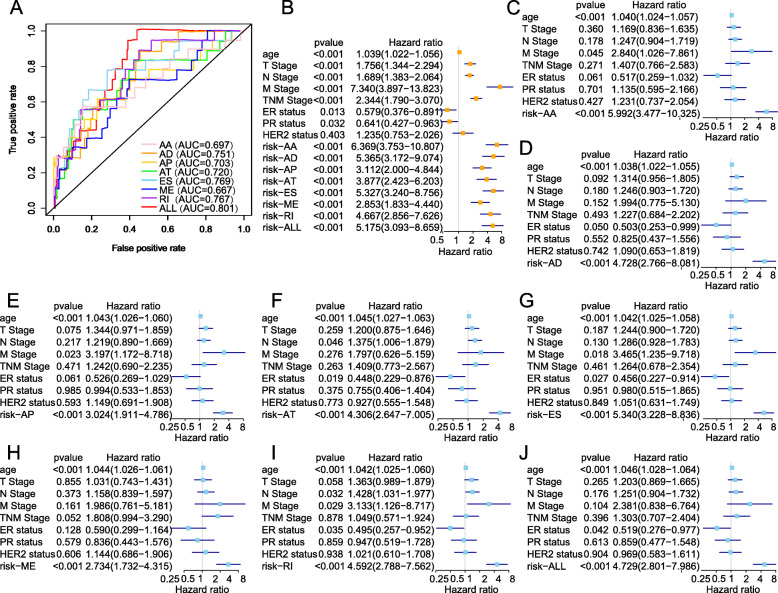
**a** ROC curves of prognostic signatures for BRCA. **b** Univariate Cox regression analysis of clinical features and prognostic signatures. **c-j** Multivariate analysis of clinicopathological features and eight prognostic signatures. **c** alternate acceptor, **d** alternate donor sites, **e** alternate promoters, **f** alternate terminators, **g** exon skips, **h** mutually exclusive exons, **i** retained introns, and **j** comprehensive signature

Figure 2a-g showed the 20 most significant prognostic AS events of each of the seven types. Because ME type accounts for the smallest percentage of AS, the only seven ME events were significant associated with survival. Seven types of prognostic signatures based on prognostic AA, AD, AP, AT, ES, ME, and RI events were developed using LASSO Cox analysis (Fig. 3a-g). Moreover, an integrated analysis of all the seven types of AS events was performed to create a comprehensive prognostic signature (abbreviated as “ALL”), which consist of PARPBP-24031-ES, NCOR1–39424-ES, COPZ1–22159-RI, ANK3–11845-AP, ITGB5–100223-ES, PHTF1–4284-RI, HSPBP1–52052-AP, TCF12–30783-AP, RPS6KA1–1282-AP, CNST-10497-ES, TMEM25–19023-AA, TMEM25–19017-AA and BTN3A2–75,630-ES (Fig. 3h, Table 1). Kaplan-Meier survival analysis showed that the eight signatures could effectively separate the survival curves of low-risk groups from those of the high-risk groups (Fig. 4a-h). Figure 5a-h showed the risk scores of eight signatures which ranked from low to high (upper panel). The median was used as a cut-off to divide high- and low-risk groups. Patients with a low-risk score had longer survival time (lower panel). Next, the efficacy of these eight prognostic signatures in prognosis prediction was evaluated by ROC curves. The area under the cure (AUC) of eight signatures was larger than 0.6. It is worth noting that the AUC of comprehensive signatures reached 0.801 (Fig. 6a). Univariate Cox regression analysis showed that the eight signatures were significantly associated with survival of patients with BRCA (Fig. 6b). Additionally, all eight signatures were identified as independent prognostic predictors for BRCA in multivariate COX analyses (Fig. 6c-j). Moreover, the correlations between ER, PR, HER2 status and prognostic risk defined by AS signatures were analyzed (Table S4). Positive ER status was associated with low risk defined by AA and ALL signatures, and high risk defined by AT signature. Positive PR status was associated with low risk defined by AA, AP, ME and ALL signatures, and high risk defined by AT signature. Positive HER2 status was associated with high risk defined by AP, AT and ALL signatures.

**Table 1:** Alternative splicing signatures associated with overall survival in patients with BRCA

AS type	Formula	HR (95% CI)	AUC
AA	(BRPF3|75960|AA×-6.16)+(TMEM25|19023|AA×-2.96)+(TMEM25|19017|AA×-2.3)+(EIF4G3|957|AA×2.01)+(SLC38A6|27787|AA×-1.91)+(PNPLA8|81412|AA×-2.33)+(SAFB|46852|AA×-3.57)+(PJA1|89360|AA×1.47)+(CAMTA2|38638|AA×-1.34)+(DBF4|80345|AA×10.15)+(SMG7|9181|AA×3.66)+(PSMD13|13632|AA×-0.61)+(ELF1|25724|AA×-22.6)+(ZNF268|25354|AA×10.09)+(SEC24D|70448|AA×3.56)+(PKMYT1|33330|AA×-1.27)+(ATXN3|28923|AA×-15.55)+(GGT5|61393|AA×-2.62)	6.369 (3.753-10.807)	0.697
AD	(POMT1|87937|AD×-1.65)+(ENOPH1|69708|AD×-3.16)+(FCF1|28425|AD×-3.4)+(DCAF11|26842|AD×-5.4)+(OBSL1|57731|AD×-4.41)+(ARSA|62901|AD×2.52)+(HNRNPUL1|50039|AD×-1.14)+(FRMD6|27515|AD×-1.89)+(RNF41|22407|AD×3.64)+(TVP23C|39359|AD×-4.64)+(FBXL12|47433|AD×2.69)+(C7orf49|81873|AD×-2.3)+(SEC31A|100880|AD×6.21)+(NKG7|51322|AD×-18.81)+(PTBP3|87243|AD×2.33)+(BCL2L11|54966|AD×-2.19)+(PDGFC|70954|AD×-8.91)	5.365 (3.172-9.074)	0.751
AP	(ANK3|11845|AP×1.14)+(HSPBP1|52052|AP×-3.16)+(RPS6KA1|1282|AP×2.6)+(NR1H3|15695|AP×-1.42)+(SH3BP2|68591|AP×1.82)+(KLF11|52654|AP×3.89)+(APOC2|50372|AP×-4.67)+(MEF2A|32713|AP×6.02)+(RAD21|84981|AP×-5.01)+(MOK|29361|AP×-3.24)+(TOP1MT|85413|AP×-9.73)+(SPAG9|42493|AP×1.79)	3.112 (2.000-4.844)	0.703
AT	(USH2A|9805|AT×-8.17)+(CYP4B1|2832|AT×-3.23)+(EYS|76614|AT×-4.42)+(DAPL1|55687|AT×-0.77)+(CDK15|56923|AT×-3.48)+(TNFRSF13B|39449|AT×-1.13)+(ATRNL1|13220|AT×1.57)+(CLEC3A|37670|AT×0.56)+(EFCAB13|42070|AT×-1.38)+(CHRNA1|56036|AT×-5.08)+(FAM184A|77360|AT×3.36)+(C3orf55|67409|AT×1.55)	3.877 (2.423-6.203)	0.72
ES	(PARPBP|24031|ES×-0.97)+(NCOR1|39424|ES×-3.52)+(UBE2V1|59762|ES×-4.81)+(HNRNPM|94942|ES×-5.83)+(ITGB5|100223|ES×-10.88)+(CNST|10497|ES×-4.78)+(SCLY|58203|ES×-4.27)+(SUV420H1|17300|ES×-7.95)+(ARHGAP12|11155|ES×-2.64)+(HARS2|73762|ES×-11.75)+(BTN3A2|75634|ES×-2.49)+(FDFT1|82653|ES×-1.36)+(RCSD1|8870|ES×-3.22)+(TDG|24084|ES×-3.41)+(FCGR2B|8681|ES×-2.17)+(BCL2L11|54967|ES×-1.75)+(ENOSF1|44469|ES×-4.23)	5.327 (3.240-8.756)	0.769
ME	(COPS5|115459|ME×1.67)+(GOLT1B|92984|ME×1.41)+(GRB10|79717|ME×-0.81)+(CPSF7|99751|ME×-1.12)+(SORBS2|71377|ME×2.69)+(SRGAP1|93242|ME×2.17)+(PTK2|98071|ME×2.21)	2.853 (1.833-4.440)	0.667
RI	(PRX|49900|RI×1.3)+(COPZ1|22159|RI×-2.86)+(PHTF1|4284|RI×1.73)+(SV2B|32540|RI×-4.98)+(CHTF8|37263|RI×-9.28)+(AK9|77206|RI×-3.77)+(GLI4|85407|RI×-1.26)+(MTF2|3771|RI×-5.62)+(PCSK7|18902|RI×-2.86)+(PDGFC|70956|RI×-9.44)+(HYAL1|64998|RI×-2.72)+(DERL3|61334|RI×1.31)+(CD59|14911|RI×-9.36)+(KIAA0895L|36957|RI×6.12)	4.667 (2.856-7.626)	0.767
ALL	(PARPBP|24031|ES×-0.91)+(NCOR1|39424|ES×-5.48)+(COPZ1|22159|RI×-2.44)+(ANK3|11845|AP×0.62)+(ITGB5|100223|ES×-22.45)+(PHTF1|4284|RI×2.17)+(HSPBP1|52052|AP×-3.02)+(TCF12|30783|AP×1.47)+(RPS6KA1|1282|AP×2.47)+(CNST|10497|ES×-3.92)+(TMEM25|19023|AA×-2.99)+(TMEM25|19017|AA×-1.08)+(BTN3A2|75630|ES×-2.65)	5.175 (3.093-8.659)	0.801

*AS* alternative splicing, *HR* hazard ratio, *AUC* area under curve, *AA* alternate acceptor, *AD* alternate donor sites, *AP* alternate promoters, *AT* alternate terminators, *ES* exon skips, *ME* mutually exclusive exons, *RI* retained introns, *ALL* all types

### Prognostic SF-AS network

**Fig. 7:**
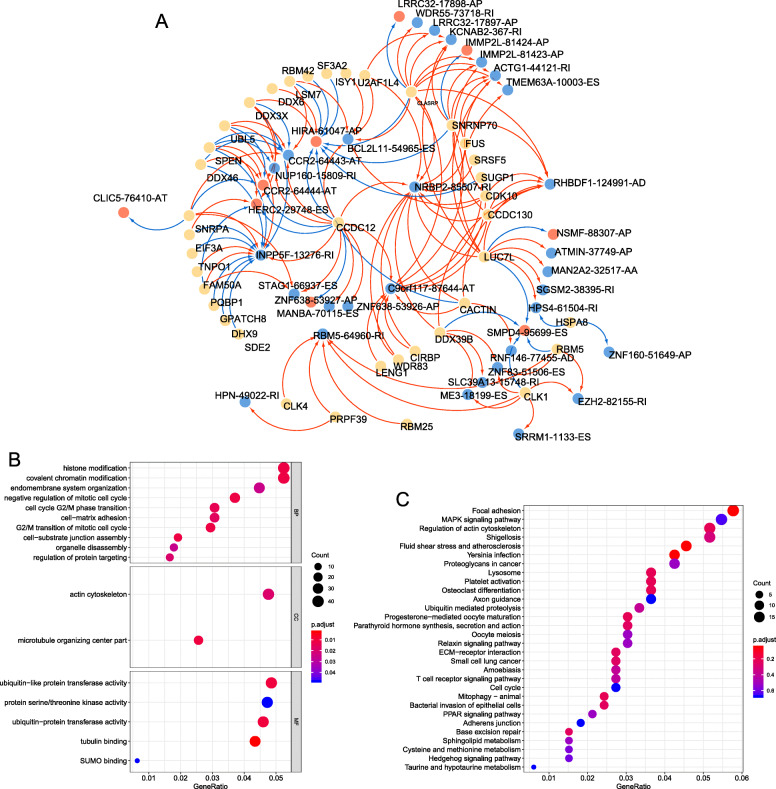
**a** Prognostic SF-AS network in BRCA. Red/blue line represents positively/negative correlation; red/blue ellipse represents risk / protective AS events; Yellow ellipse represents splicing factors. **b-c** Bubble plot displayed the GO (**b**) and KEGG (**c**) analysis of genes with prognostic alternative splicing events. BP=Biological process, CC = cellular component, MF = molecular function

The Pearson correlation analysis showed that there were 30 SFs were negatively correlated with 20 AS events, whereas 34 SFs positively correlated with 35 AS events (Table S8). A regulatory network was generated based on the correlation between AS and SFs, which consist of 29 protective AS events (associated with good prognosis), 9 risk AS events (associated with poor prognosis) and 38 SFs, (Fig. 7a). Among the SFs, CCDC12, CLASRP and LUC7L were significantly correlated with more than 10 AS events, therefore they were considered as a core SF. AS events INPP5F-13,276-RI and NRBP2–85507-RI were both regulated by more 13 SFs, they might play important roles in AS of BRCA. Moreover, the prognostic significance of the correlating SFs we analyzed. There were 8 correlating SFs (HSPA8, U2AF1L4, SNRNP70, SRSF5, CLASRP, CCDC12, SART1, and WDR83) significantly associated with survival of BRCA patients (Table S9).

### Functional enrichment analysis

The results of GO analysis suggested that AS genes were implicated in carcinogenesis associated biological processes such as “negative regulation of mitotic cell cycle”, “cell cycle G2/M phase transition”, “cell−matrix adhesion” (Fig. 7b, Table S5). In the KEGG analysis, AS genes were enriched in pathways associated with cancer, such as “MAPK signaling pathway”, “Small cell lung cancer”, and “Cell cycle” (Fig. 7c, Table S5).

## Discussion

Alternative splicing is a vital process involved in the RNA transcription and modification of mRNA isoforms [5, 15]. Increasing evidence has demonstrated that abnormal AS is associated with the carcinogenesis of multiple cancers [16–19]. Hence, exploration of AS mechanisms deepens our understanding of posttranscriptional regulatory patterns.

With the rapid development of the next-generation sequencing technology, progress has been made in the field of bioinformatics. TCGA and SpliceSeq database provides researchers with a great amount of high quality RNA sequencing data, which enabled the studies of AS patterns in various cancer types [20, 21]. To our knowledge, several studies reported AS profiles and established prognostic prediction model for several cancers, including kidney renal clear cell carcinoma [22], hepatocellular carcinoma [23], esophageal carcinoma [24], prostate adenocarcinoma [25], colorectal cancer [26], and soft tissue sarcoma [27].

Our study demonstrated that 45,421 AS events in 21,232 mRNAs were found in BRCA, and 1121 AS events in 931 genes are significantly correlated with the survival of BRCA patients. Zhang et al. identified 3071 AS events in breast cancer patients as significant for prognoses [28]. In both Zhang’s and ours studies, overall survival associated AS events were analyzed by univariate Cox proportional hazard regression analysis. In Zhang’s study, BRCA cohort were first divided into two groups (categorical variable) by a median cut of PSI value before survival analysis. In our study, we conduct Cox survival analysis with the original PSI value (continuous variable), instead of deliberately separate the cohort into two groups. Therefore, the number of identified AS events is less in our study. By integrating all seven types of AS, a comprehensive prognostic signature was generated, which included PARPBP [29], NCOR1 [30], COPZ1 [31], ANK3, ITGB5, PHTF1 [32], HSPBP1, TCF12 [33], RPS6KA1, CNST, TMEM25 [34] and BTN3A2 [35], which are play essential roles in carcinogenesis. Claudia et al. reported that PARPBP inhibits activation of the NF-κB pathway, which can initiate p21-mediated differentiation and proliferation arrest [29]. Wang et al. suggested that NCoR1 may act as tumor suppressors in GIST cells through the Smad signaling pathway [30]. Maria et al. indicated that COPZ1 represents an example of non-oncogene addiction in thyroid tumor cells, COPZ1 depletion affects thyroid tumor cell viability in vivo and in vitro [31]. Huang et al. found that PHTF1 may be a tumor-suppressor like gene and a therapeutic target for triggering the PHTF1-FEM1b-Apaf-1 apoptosis pathway [32]. Yang et al. revealed that TCF12 promotes the tumorigenesis and metastasis of hepatocellular carcinoma via upregulation of CXCR4 expression [33]. Moreover, a recent study suggested that BTN3A2 serves as a prognostic marker and favors immune infiltration in triple-negative breast cancer [35].

The comprehensive signature can serve as a useful tool to predict the survival outcomes of patients with BRCA with an AUC value of 0.801. Besides, we found there were correlation between breast cancer subtype (HER2, ER/PR) and prognostic risk defined by AS signatures. Accordingly, we speculated that HER2, ER and PR status might affect alternative splicing of certain genes that associated with cancer progression and survival outcome. More in-depth researches are warranted to provide novel insights into the molecular mechanism of BRCA. Additionally, an SFs-AS network was created and we found that CCDC12, CLASRP and LUC7L might serve as core SFs on account of their significant correlation with multiple AS events.

## Conclusions

The AS prognostic signatures accurately predict survival outcomes of BRCA patients, suggesting that AS signatures might act as ideal prognostic indicators. The SFs-AS regulatory network demonstrated the molecular mechanisms of AS in BRCA. Our study may provide potential therapeutic targets for future BRCA management.

## Supplementary Information

**Additional file 1: Table S1.** Differential AS eventsR3.

**Additional file 2: Table S2.** AS events significantly associated with OSR3.

**Additional file 3: Table S3.** List of 404 splice factorsR3.

**Additional file 4: Table S4.** ER, PR, and HER2 correlationR3.

**Additional file 5: Table S5.** Detailed results of GO and KEGG analysesR3.

**Additional file 6: Table S6.** Differential expressed genesR3.

**Additional file 7: Table S7.** AS events Ratio for 7 typesR3.

**Additional file 8: Table S8.** Correlation between AS events and SFsR3.

**Additional file 9: Table S9.** Prognostic SFsR3.

## Data Availability

All data are included in this article and supplementary files. The original data are available upon reasonable request to the corresponding author. The data of alternative splicing were downloaded from TCGA SpliceSeq database (https://bioinformatics.mdanderson.org/TCGASpliceSeq/). The expression and clinical data were downloaded from TCGA Genomic Data Commons Data Portal (https://portal.gdc.cancer.gov/). The public access to these databases is open.

## Abbreviations

BRCA: Breast cancer
AS: Alternative splicing
TCGA: The Cancer Genome Atlas
PSI: Percent Spliced In
AA: Alternate acceptor site
AD: Alternate donor site
AP: Alternate promoter
AT: Alternate terminator
ES: Exon skip
ME: Mutually exclusive exons
RI: Retained intron
OS: Overall survival
LASSO: Least absolute shrinkage and selection operator
ROC: Receiver operating characteristic
AUC: Area under the curve
SFs: Splicing factors
GO: Gene ontology
KEGG: Kyoto Encyclopedia of Genes and Genomes
BP: Biological process
CC: Cellular component
MF: Molecular function
